# Genetic control of abiotic stress-related specialized metabolites in sunflower

**DOI:** 10.1186/s12864-024-10104-9

**Published:** 2024-02-20

**Authors:** Marco Moroldo, Nicolas Blanchet, Harold Duruflé, Stéphane Bernillon, Thierry Berton, Olivier Fernandez, Yves Gibon, Annick Moing, Nicolas B. Langlade

**Affiliations:** 1 UMR LIPME, INRAE, CNRS, Université de Toulouse, 31326 Castanet Tolosan, France; 2 UMR BFP, INRAE, Université de Bordeaux, 33140 Villenave d’Ornon, France; 3 Bordeaux Metabolome, MetaboHUB, PHENOME-EMPHASIS, 33140 Villenave d’Ornon, France; 4 UMR BioForA, INRAE, ONF, Orléans, 45075 France; 5 UMR MYCSA, INRAE, 33140 Villenave d’Ornon, France; 6 USC RIBP, INRAE, Université de Reims, 51100 Reims, France

**Keywords:** Sunflower *(Helianthus annuus)*, Abiotic stresses, Metabolome, Liquid chromatography-mass spectrometry, Genome-wide association study, Antioxidants, Specialized metabolites, Reactive oxygen species

## Abstract

**Background:**

Abiotic stresses in plants include all the environmental conditions that significantly reduce yields, like drought and heat. One of the most significant effects they exert at the cellular level is the accumulation of reactive oxygen species, which cause extensive damage. Plants possess two mechanisms to counter these molecules, i.e. detoxifying enzymes and non-enzymatic antioxidants, which include many classes of specialized metabolites. Sunflower, the fourth global oilseed, is considered moderately drought resistant. Abiotic stress tolerance in this crop has been studied using many approaches, but the control of specialized metabolites in this context remains poorly understood. Here, we performed the first genome-wide association study using abiotic stress-related specialized metabolites as molecular phenotypes in sunflower. After analyzing leaf specialized metabolites of 450 hybrids using liquid chromatography-mass spectrometry, we selected a subset of these compounds based on their association with previously known abiotic stress-related quantitative trait loci. Eventually, we characterized these molecules and their associated genes.

**Results:**

We putatively annotated 30 compounds which co-localized with abiotic stress-related quantitative trait loci and which were associated to seven most likely candidate genes. A large proportion of these compounds were potential antioxidants, which was in agreement with the role of specialized metabolites in abiotic stresses. The seven associated most likely candidate genes, instead, mainly belonged to cytochromes P450 and glycosyltransferases, two large superfamilies which catalyze greatly diverse reactions and create a wide variety of chemical modifications. This was consistent with the high plasticity of specialized metabolism in plants.

**Conclusions:**

This is the first characterization of the genetic control of abiotic stress-related specialized metabolites in sunflower. By providing hints concerning the importance of antioxidant molecules in this biological context, and by highlighting some of the potential molecular mechanisms underlying their biosynthesis, it could pave the way for novel applications in breeding. Although further analyses will be required to better understand this topic, studying how antioxidants contribute to the tolerance to abiotic stresses in sunflower appears as a promising area of research.

**Supplementary Information:**

The online version contains supplementary material available at 10.1186/s12864-024-10104-9.

## Background

Abiotic stresses in plants can be defined as all the environmental conditions that decrease growth and yield below optimum levels [1]. They include, among others, drought, salinity, low and high temperatures, nutrient deficiencies, and ultraviolet radiation [2]. The impact of most of these stresses is becoming more severe because of climate change [1]. Abiotic stresses exert their effects in many complex and diverse ways, and plants have evolved a vast array of mechanisms to cope with them. Some of these mechanisms include the accumulation of wax and cutin on leaf surfaces, the desaturation of membrane lipids, and the accumulation of compatible solutes [2].

At the molecular level, one of the most significant effects of abiotic stresses is the accumulation of reactive oxygen species (ROS) [3], which arises from an imbalance between ROS production and scavenging [4, 5]. ROS are strong oxidizers and cause extensive damage to many biological molecules, like for instance proteins, lipids, and DNA [3, 5].

Plants use two mechanisms to counterbalance oxidative stress. The first is represented by detoxifying enzymes, such as superoxide dismutase, catalase, ascorbate peroxidase, and glutathione reductase [4]. The second corresponds to non-enzymatic antioxidants, i.e. ascorbic acid, reduced glutathione, α-tocopherol, and several classes of secondary or specialized metabolites such as carotenoids, flavonoids, and phenolic acids, whose ROS-scavenging activity has been demonstrated across many plant species [5, 6].

Terpenes are another class of specialized metabolites with antioxidant properties. Although better known as constituents of essential oils, allelopathic agents, and attractants or repellants in plant–herbivore interactions [7], there is increasing evidence of their implication in ROS scavenging [8–10]. Taken together, it can be stated that most specialized metabolites induced by abiotic stresses show antioxidative activity [11], which makes these molecules key players for plant adaptation to more stressful environments and for breeding tolerant varieties.

Sunflower (*Helianthus annuus* L.) is the fourth most important oilseed worldwide. It can maintain stable yields across many conditions and, largely thanks to its well-developed tap roots, it is adapted to low water-input regimes in warm to semi-arid zones [12]. Although this crop is usually considered moderately drought tolerant, the challenges posed by climate change will require major efforts in terms of breeding and crop management.

To cope more efficiently with hydric stress, tolerant varieties will have to be developed [13], and tolerance to heat will have to be jointly prioritized, because high temperatures dramatically affect pollination, fertilization, and seed set [13]. Another way to avoid drought is early sowing. This strategy allows to anticipate the timing of flowering, thus avoiding the summer periods in which evaporative demand is higher [13, 14]. However, this practice presents side-effects, because the crop is more exposed to cold stress at germination [15]. From this perspective, developing hybrids with improved tolerance to cold will be another relevant goal.

The molecular mechanisms underlying tolerance to abiotic stresses in sunflower have been studied using different approaches over the last years. Non-targeted metabolomics and proteomics have been used to profile a set of inbred lines and hybrid genotypes [16, 17] and to find biomarkers for drought tolerance [18], while transcriptome and metabolome have been integrated to identify transcription factors regulated under the same condition [19].

Transcriptome profiling has been used to describe the impact of drought using co-expression networks [20], differential analysis [21], differential analysis coupled to association genetics [22] and gene-phenotype networks [23]. It has also been chosen to characterize low-nutrient stress and three water-related stresses [24]. Eventually, tolerance to salt stress and its link with vigor have been studied through association genetics [25]. Nevertheless, to date no information is available concerning the genetic control of specialized metabolome in sunflower under abiotic stress.

In this work, we present the results of the first GWAS performed in sunflower using specialized metabolites related to abiotic stresses as molecular phenotypes. Our approach consisted of three main steps. First, we analyzed the semi-polar fraction of leaf extracts of a panel of sunflower hybrids using untargeted liquid chromatography-mass spectrometry (LC-MS), which allowed us to focus our analysis on specialized metabolites. Second, we selected a subset of these compounds based on their genetic association with some previously known quantitative trait loci (QTLs) related to yield and abiotic stress tolerance. Third, we characterized in silico these compounds and the genes associated with them.

It has also to be noticed that, in addition to our own work, GWAS using molecular phenotypes in sunflower has been used so far only in another case, i.e. to disentangle the genetic basis of oil fatty acid content [26]. Our results can therefore be considered original by a methodological point of view.

## Results

### Association mapping

To study the genetic control of specialized metabolites in sunflower and how this relates to abiotic stress tolerance, we obtained the metabolomic profiles of the leaves of 450 hybrids originating from crosses among 36 restorer and 36 cmsPET1 sterile lines grown in agronomical conditions. After the partial removal of redundancy due to isotopes and adducts, the final metabolome dataset consisted of 2557 LC-MS features (Table S01) characterized by a retention time (RT) and a mass over charge ratio (*m/z)* (Table S02). A total of 21 features already had an annotation based on previous works (Table S02) [16, 18, 27].

**Fig. 1:**
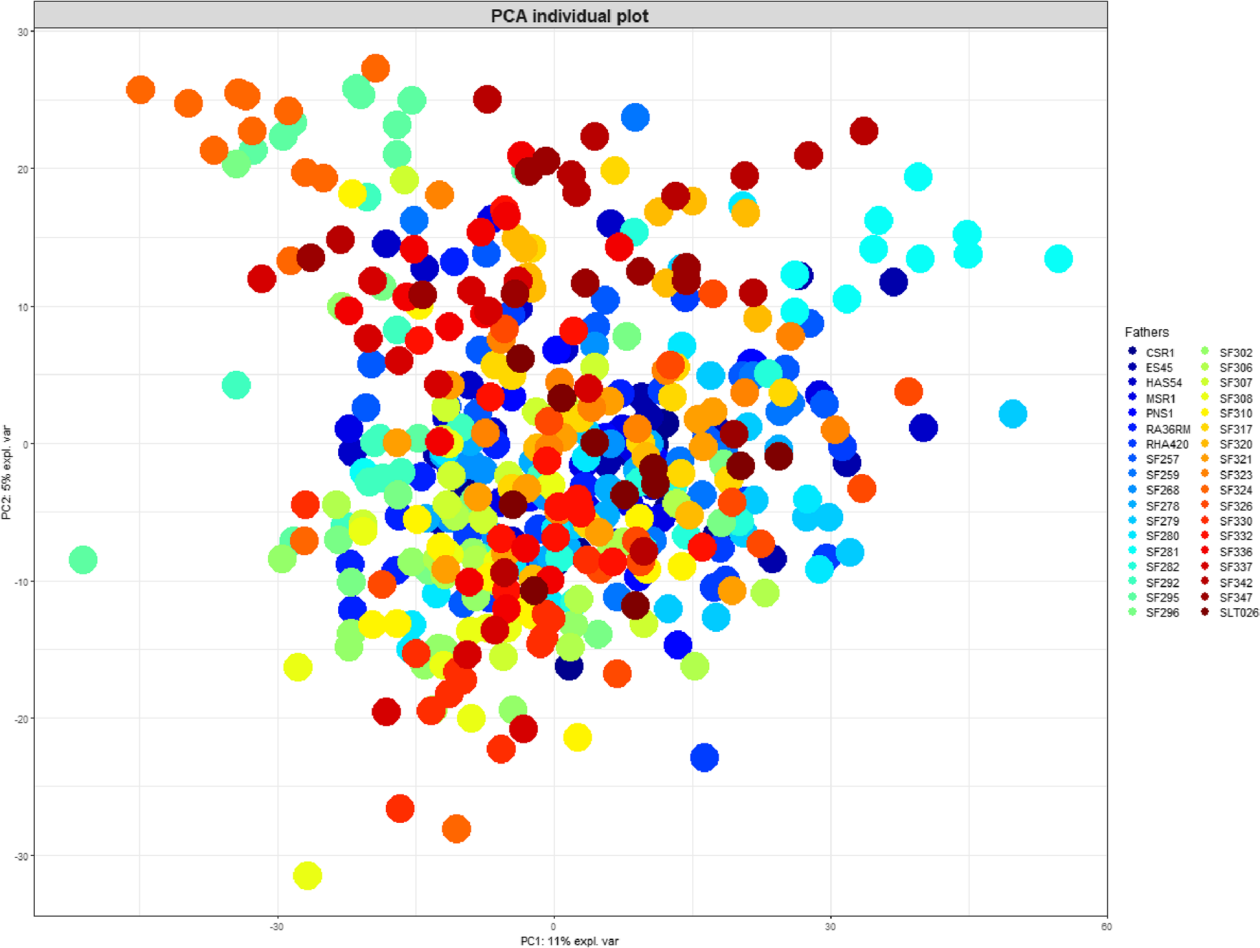
Individual plot of the first two components of the PCA based on the normalized intensities of 2557 LC-MS features measured in the leaves of 450 sunflower hybrids. The different hybrids are colored according to their male parental lines, which are indicated in the box on the right

A PCA performed using these data showed that the first two principal components accounted for 11% and 5% of total variability, which was consistent with the results obtained in similar contexts [28]. A clustering of most of the hybrids according to their male parental line was observed (Fig. 1). This was especially evident for SF295, SF324, SF330, SF342 and SF281 male lines, but could be observed in other cases as well. On the contrary, no clustering was observed according to female parental lines.

**Fig. 2:**
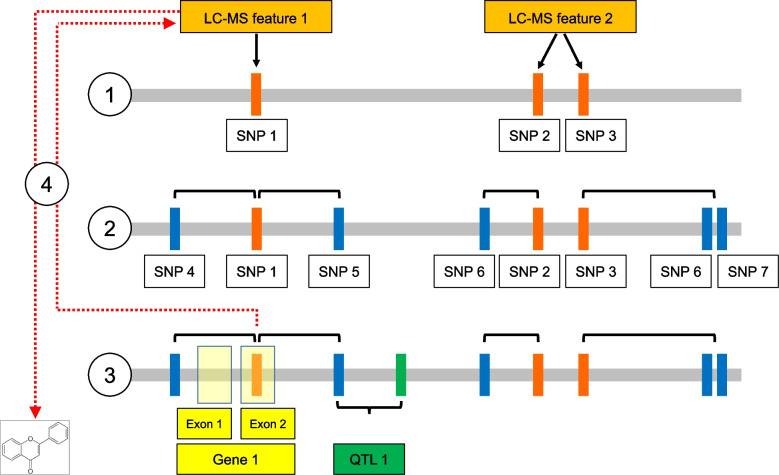
Graphical abstract illustrating the main steps of our analysis workflow. (1) First step of GWAS: detection of the associations among LC-MS features (orange boxes) and reference SNPs (red vertical bars); (2) Second step of GWAS: reference SNPs are linked to co-inherited SNPs sets (blue vertical bars); (3) Co-localization among co-inherited SNPs and SNPs belonging to abiotic stress-related QTLs (green vertical bar). Co-inherited SNPs falling in a 50 kb interval downstream or upstream of an SNP belonging to a QTL are considered co-localizing with the same QTL. All the SNPs in complete linkage disequilibrium (LD) with co-localizing SNPs and mapping to exons (yellow transparent boxes) are then used to identify putative candidate genes; (4) LC-MS features associated to reference SNPs in complete LD with co-localizing SNPs are selected and tentatively annotated

The 2557 LC-MS features were then used as an input for the first step of the association analysis, which consisted in performing GWAS using reference SNPs. A visual overview of this analysis step, as well as all the other ones included in our workflow, is found in Fig. 2. Similarly to what observed in other plant species [29], 955 LC-MS features (i.e. 37.3% of the total number used) were associated to at least one SNP. This corresponded to 2560 associations (Table S03). On average, an LC-MS feature was therefore associated to 2.7 SNPs, with 472 features associated to only one SNP and 483 features associated to two to 19 SNPs.

Among the 955 significantly associated LC-MS features, 798 (i.e. 83.6%) presented genomic heritability (h^2^_g_) values higher than 0.50, the average being 0.66 (Fig. S01), which was in line with what reported in the literature [29, 30]. The 2560 detected associations corresponded to 1716 unique reference SNPs, with 378 SNPs associated to two to 43 features. This suggested that many LC-MS features were under pleiotropic control, as already described in other species [31, 32], or that some biochemical information redundancy was still present in our data set after LC-MS data filtering. Five features already possessed an annotation based on previous works (Table S03).

The second step of association analysis consisted in linking reference SNPs to their corresponding sets of co-inherited SNPs, which included all the SNPs in complete linkage disequilibrium (LD) with them (see Fig. 2 and the Methods section). After this step, 62,134 associations were found. The number of associations per LC-MS feature ranged from one to 3666, the average being of 65.1 (Table S04). Overall, these associations corresponded to 27,246 unique SNPs.

To gather a first functional understanding of the genetic control of sunflower specialized metabolome, we then investigated all the possible associations among LC-MS features and genes in an unsupervised way, i.e. without any further biological information. A gene was considered associated to a feature if at least one SNP of a co-inherited set mapped to one of its exons (Fig. 2). Our analysis highlighted that 1768 SNPs belonging to co-inherited sets out of 27,246 (i.e. 6.5%) were found in exons.

Exonic SNPs corresponded to 533 genes, with an average of 3.3 associations per gene (Table S05). These genes appeared to be involved in several pathways, with a slight over-representation of those related to glutathione, lipids and specialized metabolite biosynthesis, like for instance flavonoids. Anyway, it must be considered that the levels of enrichment, especially in terms of numbers of genes associated to each ontology, were rather low (Table S06).

As observed in other species [29, 30], metabolite-associated SNPs and genes were not randomly distributed across the genome, but appeared to be especially concentrated in some specific ‘hot spots’ (Fig. S02). To explore this pattern of distribution, we first used a sliding window approach and subsequently, by applying a threshold based on the proportion and on the absolute number of metabolite-associated genes in each window, we defined six hot spots on chromosomes 5, 6, 7, 9, 12 and 16. These regions contained 88 metabolite-related genes and spanned 48 Mb, which corresponds to a sixth of all metabolite-related genes on slightly less than 1.5% of the sunflower genome (Table S07). In some instances, these genes were arranged in small families, like in the case of the hot spot on chromosome 5, which contained six putative quinate O-hydroxycinnamoyltransferase, and in the case of the hot spot on chromosome 6, where seven putative glutathione transferases were detected (Table S07). Anyway, despite the presence of these specific patterns, we could not find any evidence of functional metabolic clusters as defined by Nützmann and coworkers [33].

### Co-localization of SNPs associated to LC-MS features with QTLs related to abiotic stresses

As illustrated in Fig. 2, to study the genetic control of specialized metabolites linked to abiotic stresses we tested the co-localization of co-inherited SNPs associated to LC-MS features with previously identified QTL regions for drought, cold, and nutrient stress tolerance and for productivity and development-related traits [15, 34]. These last two groups of traits were added because they were considered as indirectly related to abiotic stress tolerance.

We detected a total of 638 SNPs that co-localized with 20 QTLs, among which seven were related to drought stress tolerance, five to cold stress tolerance, four to nutrient stress tolerance and four to productivity and development-related traits, with the QTLs for abiotic stress tolerances showing some overlap among them (Table S08). We then took these 638 co-localizing SNPs and searched for all the other SNPs which were in complete LD with them, and which were 10,793. On the one hand, these co-inherited SNPs were associated to 137 LC-MS features (Table S09), of which 16 were related to drought stress tolerance, 92 to cold stress tolerance, four to nutrient stress tolerance and 17 to productivity and development-related traits. A further eight LC-MS features were associated to two QTLs at the same time (Table S09). On the other hand, the same co-inherited SNPs were also associated to 155 putative candidate genes (Table S09).

### Annotation of LC-MS features of interest and identification of the most likely candidate genes

Because the LC-MS protocol used in this work did not involve data dependent MS/MS (i.e. tandem mass spectrometry; see the Methods section), the only way to perform the annotation of the 137 previously identified LC-MS features of interest was by relying on an in silico workflow. This procedure allowed to tentatively annotate 30 features, among which one was related to drought stress tolerance, 21 to cold stress tolerance, one to nutrient stress tolerance and four to productivity and development-related traits. A further three features were associated to two QTLs at the same time. Most of the annotated molecules (Table 1) belonged to the biochemical classes of terpenes (30%), flavonoids (17%), polyacetylenes (17%) and cinnamic acids (10%).

**Table 1:** Putative annotation of the 30 LC-MS features measured in the leaves of sunflower hybrids and co-localizing with QTLs of interest. All the metabolites were assigned an MSI level 3 (see Methods)

LC-MS feature	[M + H]+ m/z	RT (min)	isotopic pattern ion	putative chemical formula for [M + H]+	putative metabolite name	metabolite ID	metabolite class
M201T581	201.1637	9.69	M0	C15H21	4,5,9,10-dehydroisolongifolene isomer A	CID: 588771	sesquiterpenes
M203T379	203.1066	6.32	M0	C13H15O2	demethoxyencecalin	CID: 177040	chromenes
M287T548	287.0550	9.14	M0	C15H11O6	cyanidin	CID: 128861	flavonoids
M289T534	289.0706	8.89	M0	C15H13O6	pentahydroxychalcone	CID: 129636553	flavonoids
M307T374	307.1288	6.23	M0	C15H19O5N2	brachystemidine A	CID: 10892118	pyrroles
M349T519	349.0917	8.64	M0	C17H17O8	hexahydroxydimethylflavanone	–	flavonoids
M131T321	131.0490	5.36	M0	C9H7O	2-nonene-4,6,8-triyn-1-ol	CID: 57449462	polyacetylenes
M294T138	294.1547	2.31	M0	C12H24O7N	deoxyfructosyl-leucine	CID: 131752244	amino acid derivatives
M145T650	145.1011	10.83	M0	C11H13	1,9-undecadiene-5,7-diyne	CID: 15736690	polyacetylenes
M151T299	151.0754	4.99	M0	C9H11O2	coumaryl-alcohol isomer A	CID: 5280535	cinnamic acids
M151T342	151.0754	5.71	M0	C9H11O2	coumaryl-alcohol isomer B	CID: 5280535	cinnamic acids
M165T301	165.0545	5.02	M0	C9H9O3	coumaric acid	CID: 637542	cinnamic acids
M173T648	173.1324	10.80	M0	C13H17	1,4-tridecadiene-7,9-diyne	CID: 101410972	polyacetylenes
M175T609	175.0753	10.14	M0	C11H11O2	(2E,4E)-5-phenylpenta-2,4-dienoic acid	CID: 1549512	styrenes
M201T687	201.1637	11.45	M0	C15H21	4,5,9,10-dehydroisolongifolene isomer B	CID: 588771	sesquiterpenes
M202T649	202.1671	10.81	M1	C15H21	4,5,9,10-dehydroisolongifolene isomer C	CID: 588771	sesquiterpenes
M217T336	217.1585	5.60	M0	C15H21O	(3S,9Z)-pentadeca-9,14-dien-4,6-diyn-3-ol	CID: 163193273	polyacetylenes
M231T299	231.1378	4.99	M0	C15H19O2	dehydrocostus lactone isomer A	CID: 73174	sesquiterpenes
M231T362	231.1378	6.03	M0	C15H19O2	dehydrocostus lactone isomer B	CID: 73174	sesquiterpenes
M231T604	231.1378	10.06	M0	C15H19O2	dehydrocostus lactone isomer C	CID: 73174	sesquiterpenes
M243T272	243.0881	4.54	M0	C12H11O2N4	lumichrome	CID: 5326566	alloxazines
M247T334	247.1327	5.57	M0	C15H19O3	annuolide A	CID: 44583825	sesquiterpenes
M249T273	249.1484	4.55	M0	C15H21O3	annuolide E	CID: 131752335	sesquiterpenes
M266T572	266.1467	9.53	M1	C15H21O4	heliannuol F	CID: 10730325	sesquiterpenes
M273T341	273.1485	5.69	M0	C17H21O3	8-acetoxy-1,9,14-pentadecatriene-4,6-diyn-3-ol	CID: 14037439	polyacetylenes
M273T635_2	273.2213	10.59	M0	C19H29O	androst-5-en-4-one	CID: 22213540	steroids
M299T342	299.1278	5.70	M0	C18H19O4	3-phenyl-1-(2,3,4-trimethoxyphenyl)prop-2-en-1-one	CID: 67199147	flavonoids
M346T682	346.1001	11.37	M1	C18H17O7	tambulin	CID: 5281700	flavonoids
M372T338	372.1288	5.64	M0	C16H22O9N	4-(2-amino-3-hydroxyphenyl)-4-oxobutanoic acid glucoside	CID: 10948689	phenolic compounds
M515T343	515.2122	5.72	M0	C24H35O12	eugenol acetylrhamnosylglucoside	–	phenolic compounds

Subsequently, the results obtained from GWAS showed that 13 out of the 30 aforementioned metabolites were associated to at least one gene, corresponding to a total of 80 ‘initial’ genes (Table S10). Two metabolites, namely 1,4-tridecadiene-7,9-diyne (a polyacetylene) and 4,5,9,10-dehydroisolongifolene (a terpene), were associated to the same genes, potentially suggesting pleiotropy.

After the process of functional characterization of all of these associations, we focused on a final set of four metabolites that could be related to seven most likely candidate genes (Table 2). Three associations involved metabolites and enzyme-encoding genes, namely (i) the flavonoid pentahydroxychalcone and a member of the P450 cytochrome family; (ii) the sesquiterpene heliannuol F and a uridine diphosphate (UDP) glucosyltransferase (UGT); (iii) the flavonoid hexahydroxydimethylflavanone and three UGTs. The sesquiterpene 4,5,9,10-dehydroisolongifolene, instead, was associated to two transcription factors (TFs) of the AP2/ERF family (Table 2).

**Table 2:** List of the four tentatively annotated metabolites that were associated to seven most likely candidate genes. Column seven reports the results of the tBlastN analysis when they appear to be helpful in clarifying the already available gene descriptions

LC-MS feature	putative metabolite name	metabolite class	v2.1 gene	v2.1 gene description	tBLASTn result	co-inherited v1.0 SNP	type of mutation	base mutation	amino acid change	reference v1.0 SNP
M201T581	4,5,9,10-dehydroisolongifolene	sesquiterpenes	HanXRQr2_Chr07g0314841	putative transcription factor AP2-EREBP family	AP2/ERF transcription factor	HanXRQChr07_98876273	synonymous			HanXRQChr07_97029247
			HanXRQr2_Chr07g0314871	putative transcription factor AP2-EREBP family	AP2/ERF transcription factor	HanXRQChr07_98748814	missense	T > G	ASP>GLU	HanXRQChr07_97029247
M266T572	heliannuol F	sesquiterpenes	HanXRQr2_Chr09g0363371	putative UDP-glucuronosyl/UDP-glucosyltransferase	Glycosyltransferase 74G1, likely family 1	HanXRQChr09_10756950	missense	G > A	ALA>THR	HanXRQChr09_10756950
						HanXRQChr09_10757103	missense	G > T	ALA>SER	HanXRQChr09_10756950
M289T534	pentahydroxychalcone	flavonoids	HanXRQr2_Chr03g0130651	putative cytochrome P450	Cytochrome P450	HanXRQChr03_139760168	synonymous			HanXRQChr03_139760112
M349T519	hexahydroxydimethylflavanone	flavonoids	HanXRQr2_Chr11g0515571	putative UDP-glucuronosyl/UDP-glucosyltransferase	Glycosyltransferase 73E1, likely family 1	HanXRQChr11_157852754	missense	G > A	ASP>ASN	HanXRQChr11_157849826
						HanXRQChr11_157852773	missense	C > T	SER > LEU	HanXRQChr11_157849826
			HanXRQr2_Chr11g0515581	hypothetical protein	Glycosyltransferase 73E1, likely family 1	HanXRQChr11_157868396	missense	A > G	LYS > ARG	HanXRQChr11_157849826
						HanXRQChr11_157868891	missense	C > T	ALA>VAL	HanXRQChr11_157849826
						HanXRQChr11_157868836	missense	G > T	ASP>TYR	HanXRQChr11_157849826
			HanXRQr2_Chr11g0515591	Putative trans-zeatin O-beta-D-glucosyltransferase	Glycosyltransferase 73E1, likely family 1	HanXRQChr11_157874063	synonymous			HanXRQChr11_157849826
						HanXRQChr11_157873968	missense	C > T	TYR > HIS	HanXRQChr11_157849826
						HanXRQChr11_157874469	missense	A > G	ARG > GLY	HanXRQChr11_157849826

**Fig. 3:**
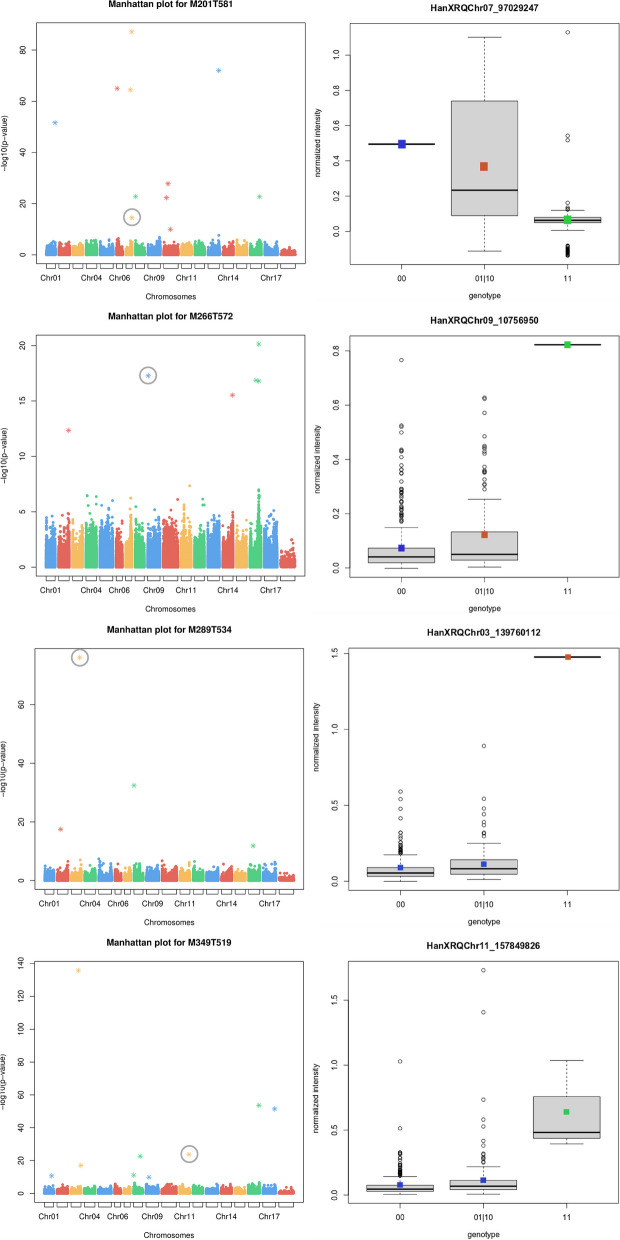
Manhattan plots and genotypic boxplots of the functionally characterized metabolites, here indicated using the codes of LC-MS features. For each metabolite, the corresponding Manhattan plot (left) and genotypic boxplot (right) are shown. Manhattan plots show the reference SNPs obtained from GWAS on the X-axis and the corresponding *p*-values on the Y-axis. SNPs filtered according to the eBIC criterion are shown as asterisks (Table S03). The reference SNP associated to a most likely candidate gene is highlighted by a grey circle. Genotypic boxplots show the normalized intensity values of the corresponding LC-MS feature (Y-axis) grouped according to the three possible allelic states (i.e. 00, 01|10, and 11). The classes identified with the Tukey’s test are indicated using colored squares. It is to note that the SNPs used to produce the genotypic boxplots are, by definition, reference SNPs, and therefore they are not the same ones found associated to the corresponding most likely candidate gene, which belong instead to co-inherited sets. The correspondences among the reference SNPs used for genotypic plots and the co-inherited used to identify most likely candidate genes are given in Table 2

Overall, these genes were linked to 13 SNPs belonging to co-inherited sets, 10 of which caused missense mutations (Table 2). Eventually, the genotypic boxplots corresponding to the four characterized metabolites showed in all of the cases a good correlation among the different allelic states and the phenotypic values of the LC-MS features (Fig. 3).

## Discussion

### Potential antioxidants represent a large proportion of sunflower leaf metabolome under abiotic stress

Oxidative damage is one of the most important modifications induced by abiotic stresses in plant cells. It is caused by the accumulation of ROS and has been reported, for instance, in the cases of drought, cold and salinity [5, 11]. Plants use many strategies to mitigate the impact of ROS, one of which is the biosynthesis of non-enzymatic antioxidants. These compounds include, among others, several important groups of semi-polar specialized metabolites like carotenoids, flavonoids, and phenolic acids [6]. Indeed, the majority of specialized metabolites produced by plants under abiotic stress show antioxidative activity in vitro, even if an in vivo experimental confirmation of their function is still lacking in many cases [11].

In this work, we studied the metabolome of 450 sunflower hybrids by performing LC-MS on the semi-polar fraction of leaf extracts, thus specifically targeting specialized metabolites. We then selected a subset of these compounds based on their genetic association to some previously known QTLs which were mainly related to abiotic stresses and, to a lesser extent, development-related traits. Eventually, we annotated this subsect of molecules. However, it must be considered that because tandem mass spectrometry had not been performed and because MS commercial standards were not available for the large majority of these metabolites, only putative annotations could be assigned.

Most of the tentatively annotated metabolites belonged to four biochemical classes, namely terpenes, flavonoids, polyacetylenes and cinnamic acids (Table 1). These findings were in line with our previous research on sunflower, and specifically with the works focusing on drought stress [16, 18]. However, the detection of polyacetylenes was unique to this study.

Flavonoids, represented by five molecules in our data, are among the best characterized ROS scavengers in plants [7, 35]. Their activity is known to provide tolerance towards many abiotic stresses, like drought [36], cold [37], and nutrient depletion [38].

Even if better known as constituents of essential oils or allelopathic agents [7], there is increasing evidence that terpenes are also involved in ROS scavenging, as described for instance in tea plant [10], sage, and rosemary [8, 9]. In other instances, the involvement of terpenes in abiotic stress tolerance has been demonstrated, even if the underlying antioxidant mechanism has not been proven yet [39].

The class of cinnamic acids, here intended as including all the derivatives of cinnamic acid, is found in most plant families, including Asteraceae [18, 27, 40]. Chlorogenic acid shows antioxidant properties, although this could be true also in the case of other cinnamic derivatives [41]. However, no ROS-scavenging activity has been demonstrated in the case of coumaric acid and coumaryl alcohol. This latter molecule, instead, is one of the precursors of lignin [42] whose accumulation, in turn, is increased for instance under drought and cold [43].

Polyacetylenes are found in a few botanical families, Apiaceae and Asteraceae being among the most relevant [44, 45]. They exhibit a wide range of antibacterial, antifungal, and insecticidal activities [45, 46].

The remaining annotated compounds belonged to several different classes. Phenolic acids, represented by 4-(2-amino-3-hydroxyphenyl)-4-oxobutanoic acid glucoside and eugenol acetylrhamnosylglucoside, are well characterized as antioxidants in plants [6, 47].

The mammal steroid androstenone is found in many plant species [48]. Mammal steroids in plants are known to be involved in processes such as root and shoot growth [49], but no information is specifically available for androstenone.

Demethoxyencecalin is a chromene. The compounds in this class have been described in plants such as mulberry [50] and *Hypericum polyanthemum* [51], but they are especially frequent in Asteraceae [52]. They are repellent towards herbivorous insects.

(2E,4E)-5-phenylpenta-2,4-dienoic acid belongs to styrenes, which are naturally synthesized for instance by mulberry [50] and styrax [53]. Some styrenes show antifeedant activity against insects in pear [54], but no evidence is available in the case of (2E,4E)-5-phenylpenta-2,4-dienoic acid.

The last three molecules highlighted by our study are lumichrome (i.e. an alloxazine), brachystemidine A, (i.e. a pyrrole), and deoxyfructosyl-leucine (i.e. an amino acid derivative). Their role is not clear and therefore difficult to discuss in our biological context.

Altogether, it is possible to affirm that a relevant fraction of the metabolites that we have tentatively annotated, i.e. from seven (considering flavonoids and phenolic acids) to 16 (including also terpenes) out of 30, fit in biochemical classes with oxygen scavenging properties.

Although the number of characterized molecules in our work is relatively small, and even if their specific in vivo activity has not been proven yet, our findings can be considered in agreement with the high proportion of antioxidants observed by many authors in abiotic stress-related specialized metabolome [11, 31, 55, 56].

### The most likely candidate genes mainly belong to two highly diverse superfamilies

Interestingly, the five enzyme-encoding most likely candidate genes found in our study belonged to only two families, namely cytochromes P450 (CYPs) and glycosyltransferases (GTs). Although very different in their functionalities, both of them are large and catalyze greatly diverse reactions that create a vast array of chemical modifications. This is consistent with the high level of plasticity of specialized metabolism pathways in plants [57].

The reactions catalyzed by CYPs play a basic role in defining the skeletal structure of many metabolites, such as flavonoids and terpenes [58, 59]. These reactions include hydroxylations, reductive activations, ring couplings, ring formations, ring expansions and oxidative aryl migrations [60, 61].

Today, a large number of CYPs involved in flavonoid biosynthesis are known in many plant families, including Asteraceae [62, 63]. It is therefore possible to hypothesize that the enzyme encoded by the *HanXRQr2_Chr03g0130651* gene performs one of the molecular reactions that lead to the biosynthesis of the flavonoid pentahydroxychalcone.

Unlike cytochromes P450, all glycosyltransferases catalyze the same type of reaction, i.e. the transfer of a sugar moiety to an acceptor. Anyway, they act on a broad range of compounds such as lipids, proteins, nucleic acids and other molecules [64].

GTs are classified in many families according to the CAZy database (http://www.cazy.org/). Family 1 is defined by the presence of a specific domain [64, 65] and is usually referred to as UDP-glycosyltransferases (UGTs). Plant UGTs are especially involved in the glycosylation of specialized metabolites such as flavonoids and terpenes [66].

Glycosylation increases the activity and the availability of both these categories of compounds and hence the ROS-scavenging capacity of the plant, which confers tolerance to several abiotic stresses. In *Arabidopsis*, for instance, the enzymes encoded by *UGT79B2* and *UGT79B3* add a UDP-rhamnose to the flavonoids cyanidin and cyanidin 3-O-glucoside. An increased concentration of these two molecules provides tolerance to cold, salinity and drought [67]. In tea plant, instead, *CsUGT78A14–1* and *CsUGT78A14–2* are involved in the biosynthesis of the flavonoids kaempferol 3-O-glucoside and kaempferol diglucoside, which reduces oxidative damage and increases tolerance to cold stress [68]. Another similar example is provided by the action of sesquiterpene nerolidol in tea plant. Again, this metabolite is glycosylated by the protein encoded by *CsUGT91Q2*, which causes an enhanced level of tolerance against cold stress [10].

In light of this, it could be speculated that the three UGTs associated to the flavonoid hexahydroxydimethylflavanone, i.e. *HanXRQr2_Chr11g0515571*, *HanXRQr2_Chr11g0515581* and *HanXRQr2_Chr11g0515591*, could be involved in the glycosylation of this molecule, thus contributing to reduce the impact of oxidative damage in sunflower. Likewise, the UGT *HanXRQr2_Chr09g0363371* could be involved in ROS scavenging through the glycosylation of the terpene heliannuol F.

Besides enzyme-encoding genes, we also found that two transcription factors of the AP2/ERF family, i.e. *HanXRQr2_Chr07g0314841* and *HanXRQr2_Chr07g0314871* were associated to the terpene 4,5,9,10-dehydroisolongifolene. Because some transcription factors of this family are implicated in the biosynthesis of terpenes, as in the cases of orange [69] and *Litsea cubeba* [70], it is possible to imagine a potential link with the aforementioned metabolite.

As already stated, 4,5,9,10-dehydroisolongifolene was associated to the same co-inherited SNPs sets that were linked to the polyacetylene 1,4-tridecadiene-7,9-diyne. This could suggest a potential case of pleiotropic control of metabolite biosynthesis, which has already been described in other plants [31, 32]. Because the biochemical pathways of terpenes and polyacetylenes are completely different, the only genes that could explain this case of pleiotropy are indeed the previously indicated AP2/ERF transcription factors. Anyway, to date information about which TFs could be involved in the biosynthesis of polyacetylenes is lacking, thus making it difficult to draw conclusions in this respect.

Despite the limited number of most likely candidate genes identified, our results appear globally in agreement with those obtained from similar metabolic GWAS analyses performed under abiotic stress in *Arabidopsis thaliana* [71] and maize [72].

It has also to be considered that our capability to identify the most likely candidate genes was reduced by some technical limitations and specific features of our study. On the one hand, gene functions are largely unknown in plants, particularly in a species such as sunflower. Indeed, 12 genes out of the 80 that were initially found associated with co-inherited SNPs sets, i.e. previous to the process of functional characterization, were annotated as ‘hypothetical’ or ‘putative’ proteins. On the other hand, our approach to link SNPs to genes was rather stringent, because it required the SNPs to directly land on exons in order to identify a potential candidate.

## Conclusions

Our work represents the first characterization of the genetic control of abiotic stress-related specialized metabolites in sunflower. It provides hints concerning the importance of antioxidant compounds in this biological context, and it highlights some of the potential molecular mechanisms underlying their biosynthesis, thus paving the way for novel applications in sunflower breeding. Even if our capability to identify candidate genes was diminished by some technical limitations, our results were consistent with those obtained from similar metabolic GWAS performed under abiotic stress in plants such as *Arabidopsis thaliana* and maize. Although further analyses will be needed to obtain a deeper understanding of the topic, studying how antioxidants contribute to the tolerance to abiotic stresses in sunflower appears as a promising area of research.

## Methods

### Plant material and sampling

A panel of 475 sunflower hybrids, corresponding to an incomplete factorial design, was obtained by crossing 36 male and 36 female inbred lines as previously described [73, 74]. Each hybrid was named using its respective female and male parental lines and adding an underscore to separate them.

As already described [75], each hybrid was grown in a single 13 m^2^ plot, and all the plots were cultivated on the same field trial in Anais (Charente-Maritime, France) from 2 May 2015 to 29 September 2015. Four control hybrids, corresponding to 65 plots, were included in the field trial to allow for the subsequent adjustment of spatial biases. Therefore, the trial included a total number of 540 plots.

For each single plot, *n*-4 topmost leaves without petioles were sampled from four different plants on July 22 2015, i.e. 7 days (± 3 days according to genotypes) after blooming, between 11:00 to 12:30 (CET time), and then pooled. Each pool was immediately frozen in dry ice and stored at − 80 °C until grinding.

### Metabolome profiling

Leaf samples were cryoground using a Retsch Mill MM 400 ball mixer (Thermo Scientific, Waltham, MA, USA) and lyophilized. Aliquots of 10 ± 1.0 mg of dry leaf powders were weighed in 1.1 mL Micronic tubes (Micronic, Lelystad, The Netherlands) and extracted at room temperature with a robotized Star/Starlet platform (Hamilton, Reno, NV, US) using ethanol/water (80:20, v/v) added with 0.1% formic acid and 1.37 mM methyl vanillate as solvent. Methyl vanillate was used as internal standard to verify the quality of injection for LC-MS.

Two successive extractions (1 min shaking followed by 15 min ultra-sonication) were performed with 300 μL of extraction solvent. The two supernatants were combined and filtered using 0.22 μm hydrophilic Durapore filtering microplates (Merck Millipore, Carrigtwohill, Ireland). Several blank extracts were prepared using the same procedure and without sample powder. A quality control (QC) sample was prepared by pooling 10 μL of each sample extract.

LC-MS profiling was performed using the ethanol supernatant extracts. The sample injection order was randomized, and QC samples were injected every 10 samples to correct for the signal intensity drift. The extracts were analyzed using an LTQ Orbitrap Elite mass spectrometer (Thermo Scientific) interfaced to an UltiMate 3000 L UHPLC system (Thermo Scientific) using a C18 chromatographic column (C18-Gemini 2.0 × 150 mm, 3 μm, 110 Å, Phenomenex, Torrance, CA, USA). An 18-min acetonitrile gradient in acidified water (solvent A: ultrapure water + 0.1% formic acid, solvent B: LC-MS grade acetonitrile) was used with a 300 μL/min flow rate and the following elution gradient: 0–0.5 min, 3% B; 0.5–1 min, 3–10% B; 1–9 min, 10–50% B; 9–13 min, 50–100% B; 13–14 min, 100% B; 14–14.5 min 100–3% B; 14.5–18 min, 3% B. The column temperature was set at 30 °C and the injection volume was 5 μL. The LC-MS instrument was equipped with an electrospray ionization (ESI) source operated in the positive ion mode. Source parameters were set as follows: source voltage, 3.2 kV; sheath gas, 45 arbitrary units (a.u.); auxiliary gas, 15 a.u.; sweep gas, 0 a.u.; capillary temperature, 350 °C; heater temperature, 350 °C. Full scan MS spectra were acquired at 240 k resolution power at 200 *m/z* with a 50–1000 *m/z* range. All the chemicals used for LC-MS were purchased from Sigma Aldrich (Saint Louis, MO, USA) and Extrasynthese (Genay, France).

LC-MS data were processed using the ‘XCMS’ R package [76]. Variables detected in blank extracts, with *m/z* values varying by more than 0.005 Da or with RT varying by more than 40 s between different samples were filtered out. Variables with intensity coefficients of variation in QCs greater than 20% were also removed. This resulted in a matrix of 3507 metabolite features. Intensity drift was corrected using support vector regression [77], and intensities were normalized according to the sample powder mass used for extraction. After a final step of quality assessment, the LC-MS data corresponding to 450 hybrids and 64 control hybrids were retained.

### Processing, annotation and exploratory analysis of metabolome data

Biases occurring because of the spatial variation in the field trial were adjusted based on the information obtained from randomly replicated control hybrids using a script based on the ‘ASReml-R’ R package v 3.0 [78]. Data from control hybrids were discarded and the spatially corrected matrix was used for the subsequent steps of analysis.

Isotopes and adducts were searched for among the initial 3507 LC-MS features using the ‘Binner’ software [79]. A total of 950 redundant variables were removed, thus bringing the final LC-MS dataset to 2557 features. The most intense ions were then annotated using RT and accurate *m/z* values and the information available from previous studies [16, 18, 27]. This resulted in the putative annotation of 21 compounds (Table S02), whose MSI levels were attributed according to [80]. Eventually, to gather information about the structure of metabolome data, a PCA was carried out after scaling and mean centering using the ‘pca’ function of the ‘mixOmics’ R package v 6.16.3 [81].

### Genotyping

The genotyping of the hybrids was carried out within the frame of the sunflower genome sequencing project [74]. Briefly, the parent lines were genotyped by whole genome resequencing, and the genotype of each hybrid was then obtained from those of its parents [74].

Initially, 14,127,553 SNPs were detected using the XRQ v1.0 assembly of the sunflower genome. Then, all the sets of SNPs in complete linkage disequilibrium among them, called ‘co-inherited SNPs sets’, were identified, and only one SNP was kept for each set. Subsequently, SNPs presenting a minor allelic frequency (MAF) < 0.1 or only detected in the male or female panel were filtered out. A final number of 350,052 SNPs, referred to as ‘reference SNPs’, were used for GWAS and are available through the Heliagene XRQ v1.0 genome portal (https://www.heliagene.org/HanXRQ-SUNRISE/). The genotypes of hybrids were coded as ‘0’, ‘1’, or ‘2’ for homozygous XRQ, heterozygous and variant homozygous, respectively. Both the additive (A) and the dominant (D) centered genotyping matrices were produced [73].

### Association analysis

Association analysis was performed in two steps (Fig. 2). First, a multi-locus with forward selection GWAS was carried out with the ‘mlmm.gwas’ R package v 1.0.6 [82] and using the 2557 filtered LC-MS features and the 350,052 reference SNPs. Both the additive (A) and the dominant (D) effects of SNP markers were considered and 20 maximum steps were imposed for the fitting of the linear mixed model, which had an equation of this form:

$${y}_i=\mu +{x}_i^l{\theta}_a^l+{w}_i^l{\theta}_d^l+{A}_i+{D}_i+{e}_i$$

Where ${x}_i^l$ is the centered genotype of the *i*^th^ hybrid at the *l*^th^ marker locus; ${w}_i^l$ is defined later; ${\theta}_a^l$ is the additive effect of the *l*^th^ locus; ${\theta}_d^l$ is the dominance effect of the *l*^th^ locus; and *e*_*i*_ denotes error.

*A*_*i*_ is the random additive effect of the *i*^th^ hybrid with the vector A ∼ $\mathcal{N}$ (*0*, ${\sigma}_a^2{K}_a$), *D*_*i*_ is the random dominant effect of the *i*^th^ hybrid with the vector D ∼ $\mathcal{N}$ (*0*, ${\sigma}_d^2{K}_d$), *e*_*i*_ is the residual error of the *i*^th^ hybrid with the vector e ∼ $\mathcal{N}$ (*0*, ${\sigma}_e^2 Id$) and *Id* the identity matrix. *K*_*a*_ is the additive and *K*_*d*_ is the dominance kinship matrix calculated using the alike in state (AIS) relatedness criterion as indicated by [83]; ${\sigma}_a^2$, ${\sigma}_d^2$ and ${\sigma}_e^2$ are additive, dominance and residual variances, respectively; and ${w}_i^l$ is calculated as already described [83].

The best GWAS model was chosen using the extended Bayesian information criterion (eBIC) as proposed by [84]. The value of genomic heritability (h^2^_g_) for each LC-MS feature was calculated using the general purpose solver function ‘mixed.solve’ of the ‘rrBLUP’ R package v 4.6.1 [85].

The second step of the analysis consisted in linking reference SNPs to their corresponding co-inherited SNPs sets, which included all the SNPs in complete linkage disequilibrium with them (Fig. 2). This step was similar to the ‘block analysis’ conducted by Temme and coworkers [25], although in our case blocks were defined by requiring complete LD among the different SNPs.

### Unsupervised identification and enrichment analysis of putative candidate genes

To identify the candidate genes putatively involved in the biosynthesis of metabolites in an unsupervised way, i.e. without any further biological information, SNPs from co-inherited sets were mapped to exons, introns, and intergenic regions using a gft file corresponding to the annotation of the XRQ v1.0 sunflower genome (http://www.heliagene.org/HanXRQ-SUNRISE/). A gene was then considered associated to an LC-MS feature if at least one SNP from a co-inherited set mapped to one of its exons. The corresponding XRQ v2.1 genes were identified using the synonymy table available at the ‘Download’ section of the XRQ v2.1 portal at (https://www.heliagene.org/HanXRQr2.0-SUNRISE/).

The putative candidate XRQ v2.1 genes were used to perform enrichment analyses using the software ClueGO 2.5.8 [86]. A two-tailed hypergeometric test was performed to identify enriched ontology terms. Significance was set at a Benjamini-Hochberg-adjusted *p*-value of 0.05, the ‘GO fusion’ option was used and the k-score was fixed at 0.4. Three custom sunflower ontologies were used for the analysis, corresponding to the two Gene Ontology (GO) subsets ‘biological process’ and ‘molecular function’ and to the KEGG pathways.

The GO sub-ontology files were built using the Blast2GO output files available on the Heliagene website (https://www.heliagene.org/HanXRQr2.0-SUNRISE/), while the KEGG ontology file was created by performing a double best hit search using the XRQ v2.1 sunflower protein sequences on the KAAS automatic annotation server (https://www.genome.jp/kegg/kaas/). The KO codes thus obtained were then manually inspected in order to remove ontologies spuriously related to bacteria, fungi and animals.

### Hot spots of metabolite-associated SNPs and genes

To describe the patterns of localization of metabolite-associated SNPs and genes along the sunflower genome and detect the potential presence of hot spots, a sliding windows approach was chosen. The window length was set at 5 Mb, and the window was incrementally advanced along the chromosomes using a pass of 1 Mb. For each window, the following measures were calculated: (i) the absolute number of metabolite-associated SNPs and their frequency respect to the total number of SNPs, and (ii) the absolute number of metabolite-associated genes and their frequency respect to the total number of genes. The values of the frequencies thus obtained were then plotted against the sunflower chromosomes and visualized with the ‘Circlize’ R package v 0.4.14 [87].

A sliding window was considered as being part of a hot spot if it presented a frequency of metabolite-associated genes higher than 0.075 and an absolute number of genes higher than 10, and adjacent windows were merged in order to obtain the final hot spots. Eventually, the identification of potential metabolic clusters as defined by [33] was performed by visually inspecting the identified regions.

### Identification of the SNPs co-localizing with known abiotic stress-related QTLs and of the associated LC-MS features

To study the genetic control of specialized metabolites linked to abiotic stresses, we followed a strategy based on co-localization with QTLs of interest that had been discovered in prior works. First, SNPs from co-inherited sets obtained from our GWAS analysis were tested for co-localization with two groups of QTLs, i.e.: (i) QTLs related to drought, cold, and nutrient stress tolerance which had been detected on other field trials; (ii) QTLs related to productivity and development traits which had been detected either on the same field trial or on other trials [15, 34]. An SNP was considered as co-localizing with a QTL if it fell in an interval of 50 kb downstream or upstream respect to an SNP belonging to the QTL itself. Second, all the SNPs in complete LD with co-localizing SNPs were identified and subsequently used to identify the putative candidate genes for metabolite biosynthesis. Eventually, using the previously defined associations among co-inherited SNPs and reference SNPs, reference SNPs co-localizing with QTLs were found. These reference SNPs were then used to determine which LC-MS features could be considered as co-localizing with QTLs, and which therefore had to be annotated.

### Annotation of LC-MS features co-localizing with known QTLs

None of the LC-MS features co-localizing with known QTLs possessed an annotation based on previous works (see previous paragraph). Therefore, the tentative annotation of these features was based on their raw chemical formulas and on the comparison with MS-related information available from KNApSAcK (http://www.cheminfo.org/Chemistry/Database/Knapsack/index.html) and Dictionary of natural products (https://dnp.chemnetbase.com). This resulted in the putative annotation of 30 other compounds belonging to 11 compound families (Table 1).

### Identification of the most likely candidate genes

The putative candidate genes previously identified through GWAS and co-localization analysis were further characterized in order to identify the most likely candidates for the associations with putative metabolites. This was carried out in a functional perspective and following a two-step procedure. The first step focused on the annotated metabolites and consisted in a systematic bibliographic search through the NCBI PubMed database (https://pubmed.ncbi.nlm.nih.gov). The names of the metabolites and of the corresponding biochemical families were used as inputs.

The second step focused on the genes, and consisted in (i) retrieving the gene descriptions available for each gene using a gft file corresponding to the XRQ v2.1 of the sunflower genome; (ii) carrying out tBLASTn searches through the NCBI BLAST website (https://blast.ncbi.nlm.nih.gov/Blast.cgi); (iii) performing a systematic bibliographic search through the NCBI PubMed database, using both the gene descriptions and the gene names obtained with the tBLASTn search as inputs.

Genotypic boxplots were produced for all the reference SNPs associated to the tentatively annotated LC-MS features using the ‘genotypes.boxplot’ function of the ‘mlmm.gwas’ R package v 1.0.6. Eventually, we characterized all the co-inherited SNPs found in exons by determining if they represented synonymous or missense mutations using the information available for the XRQ v1.0 sunflower genome (http://www.heliagene.org/HanXRQ-SUNRISE/).

### Supplementary Information

**Supplementary Material 1.**

**Supplementary Material 2.**

**Supplementary Material 3.**

**Supplementary Material 4.**

**Supplementary Material 5.**

**Supplementary Material 6.**

**Supplementary Material 7.**

**Supplementary Material 8.**

**Supplementary Material 9.**

**Supplementary Material 10.**

**Supplementary Material 11.**

**Supplementary Material 12.**

## Data Availability

The genotype and the kinship matrices needed to perform GWAS and the information concerning previously known QTLs are found in the corresponding cited references. All the genomic information concerning candidate genes are available on the Heliagene portal (http://www.heliagene.org). LC-MS data are reported in Tables S01-S02. The results of all the other analyses are reported in Tables S03-S10 and in Figs. S01-S02.

## Abbreviations

ROS: Reactive oxygen species
LC-MS: Liquid chromatography coupled to mass spectrometry
RT: Retention time
*m/z*: Mass over charge ratio
SNP: Single nucleotide polymorphism
GWAS: Genome-wide association study
PCA: Principal component analysis
QTL: Quantitative trait locus
LD: Linkage disequilibrium
