# Supplementary material for: Genetic control of abiotic stress-related specialized metabolites in sunflower
Source: BMC Genomics. 2024 Feb 20;25:199. doi: 10.1186/s12864-024-10104-9 (PMC10877922; doi:10.1186/s12864-024-10104-9)
Supplement: Supplementary file 1 — Supplementary Material 1. [file 12864_2024_10104_MOESM1_ESM.pptx]

## Slide 1
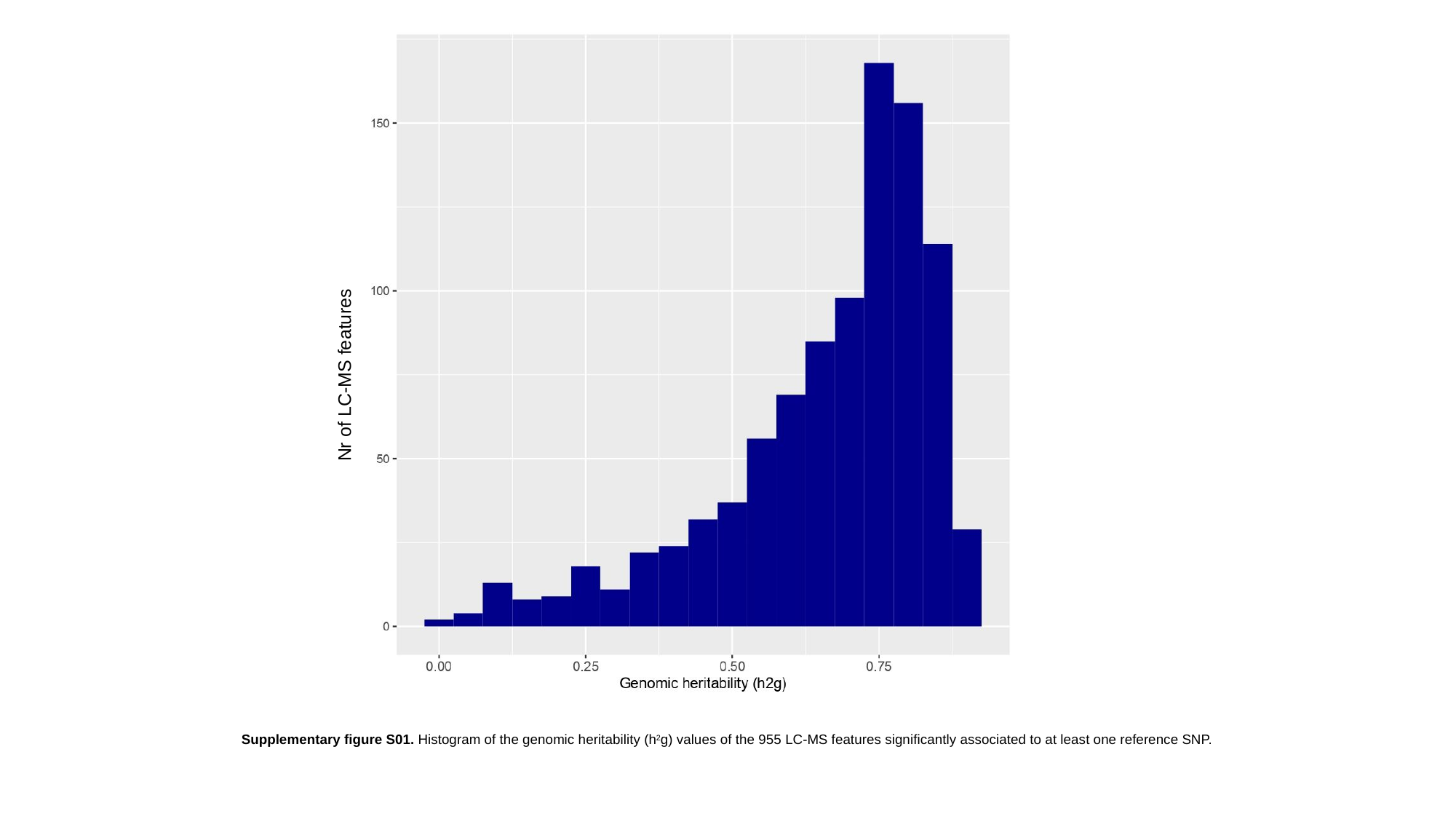

Nr of LC-MS features
Supplementary figure S01. Histogram of the genomic heritability (h2g) values of the 955 LC-MS features significantly associated to at least one reference SNP.
