# Supplementary material for: Genetic control of abiotic stress-related specialized metabolites in sunflower
Source: BMC Genomics. 2024 Feb 20;25:199. doi: 10.1186/s12864-024-10104-9 (PMC10877922; doi:10.1186/s12864-024-10104-9)
Supplement: Supplementary file 2 — Supplementary Material 2. [file 12864_2024_10104_MOESM2_ESM.pptx]

## Slide 1
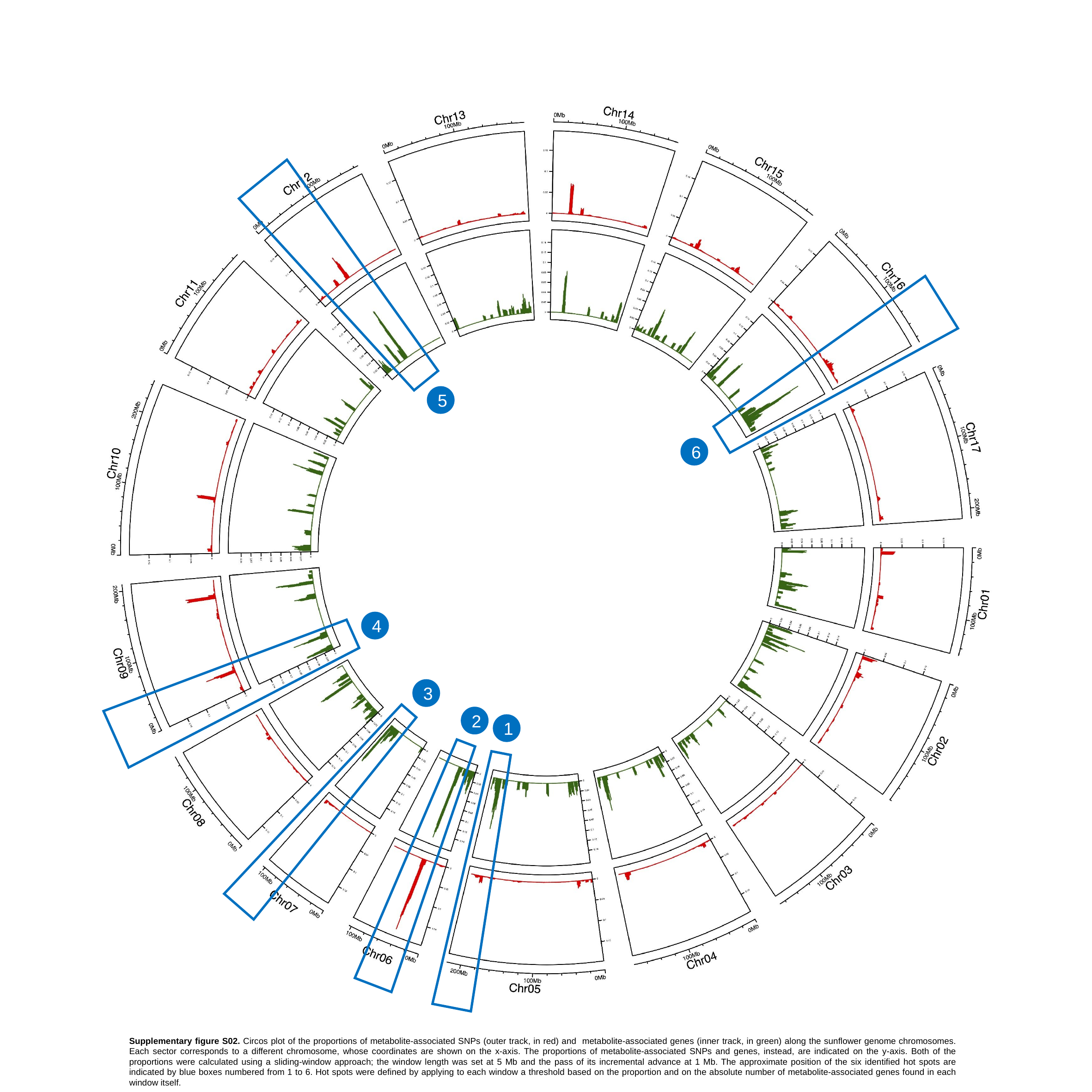

5
6
4
3
2
1
Supplementary figure S02. Circos plot of the proportions of metabolite-associated SNPs (outer track, in red) and metabolite-associated genes (inner track, in green) along the sunflower genome chromosomes. Each sector corresponds to a different chromosome, whose coordinates are shown on the x-axis. The proportions of metabolite-associated SNPs and genes, instead, are indicated on the y-axis. Both of the proportions were calculated using a sliding-window approach; the window length was set at 5 Mb and the pass of its incremental advance at 1 Mb. The approximate position of the six identified hot spots are indicated by blue boxes numbered from 1 to 6. Hot spots were defined by applying to each window a threshold based on the proportion and on the absolute number of metabolite-associated genes found in each window itself.
